# Death as rebirth: how efferocytosis drives tissue repair and disease treatment

**DOI:** 10.3389/fimmu.2025.1712547

**Published:** 2026-01-09

**Authors:** Xinliang Duan, Sichen Han, Yifu Bian, Yao Yuan, Jiayu Shen, Yixuan Dai, Junzi Mi, Zilin Wang

**Affiliations:** 1 Department of Oral and Maxillofacial Surgery, School and Hospital of Stomatology, Jilin University, Changchun, China; 2 Jilin Provincial Key Laboratory of Tooth Development and Bone Remodeling, Jilin University, Changchun, China

**Keywords:** apoptotic cells, atherosclerosis, efferocytosis, immune regulation, neural regeneration, tissue repair

## Abstract

Efferocytosis is a crucial process by which apoptotic cells are removed by phagocytes such as macrophages. It not only maintains tissue homeostasis and immune tolerance, but also plays a central role in tissue repair and disease regulation. In recent years, dysfunction of efferocytosis has been found to be closely related to various diseases, such as atherosclerosis, cancer, and chronic inflammatory conditions. Activating or remodeling efferocytosis has become a key direction in regenerative medicine and precision therapy. This review systematically summarizes the molecular mechanisms and immunomodulatory functions of efferocytosis, highlights its roles in various models of tissue injury and repair, and explores recent advances in targeted modulation of efferocytosis using nanomaterials, biomaterial scaffolds, and hydrogels. By focusing on the biological logic of “life from death,” this article aims to provide a theoretical foundation and strategic support for the translational applications of efferocytosis in tissue engineering, immunotherapy, and disease intervention. Furthermore, it highlights the synergistic potential of combining nanomaterials, hydrogels, and biological scaffolds to create next-generation efferocytosis-driven therapies, thereby offering a multidisciplinary perspective on overcoming current challenges in clinical translation. Specifically, this synergistic strategy holds particular promise in addressing key clinical challenges such as overcoming tumor immunosuppression and promoting vascularization in chronic wounds, providing a targeted approach to enhance therapeutic outcomes in these complex pathological contexts.

## 1 Introduction

Cell death does not mark the end of life but represents a key phase in the maintenance and regeneration of tissue homeostasis (1). In biomedical research, efferocytosis, a tightly regulated mechanism for clearing apoptotic cells, plays essential roles in apoptotic clearance, immune balance, and tissue repair (2). As a conserved and spatial-temporally precise process, efferocytosis is not only critical for the daily clearance of large numbers of apoptotic cells in the body, but also deeply involved in physiological and pathological processes by suppressing inflammation, promoting tissue repair, and maintaining immune tolerance (3).

With deeper investigation, efferocytosis is no longer seen as a mere scavenging behavior but rather as a complex biological network that regulates local immune homeostasis, modulates the tissue microenvironment, and mediates regenerative processes. Dysregulation of efferocytosis has been identified as a key mechanism in diseases such as atherosclerosis (4), tumor immune evasion (5), and rheumatoid arthritis (6). Conversely, moderate activation of efferocytosis can reshape immune environments, promote tissue regeneration, and reverse disease progression (7). These insights are driving the shift of efferocytosis from basic research to translational application, offering new therapeutic avenues.

Meanwhile, with the advancement of nanomaterials, hydrogels, and biomaterial scaffolds, researchers have begun to integrate these tools with efferocytosis regulation to develop novel therapeutic strategies characterized by strong targeting ability, minimal side effects, and well-defined mechanisms. This “life-from-death” strategy, by regulating the clearance of dying cells, activates pathways of tissue regeneration and immune modulation, and has become a promising direction in precision medicine. However, the role of efferocytosis is not universally beneficial. While it is essential for tissue homeostasis, dysregulated or excessive efferocytosis may also contribute to pathological outcomes such as fibrosis, immune evasion, or sustained inflammation. For instance, in advanced tumors, efferocytosis by tumor-associated macrophages can suppress antitumor immunity and promote metastasis, highlighting the context-dependent nature of this process (8). These dualities underscore the importance of understanding microenvironmental cues that determine whether efferocytosis promotes resolution or exacerbates disease. Notably, recent advances in cancer immunotherapy have highlighted the promising strategy of combining immune checkpoint inhibitors (ICIs) with therapeutic cancer vaccines. Emerging evidence suggests that the co-administration of anti-checkpoint agents (e.g., PD-1/CTLA-4 blockers) and cancer vaccines can synergistically enhance antigen-specific T cell responses, reverse immunosuppression in the tumor microenvironment (TME), and thereby improve antitumor efficacy (9). In this context, efferocytosis—a key process regulating immune homeostasis in the TME—directly influences the development of vaccine-induced immunological memory and the therapeutic response to checkpoint blockade. Therefore, integrating efferocytosis-modulating strategies with cancer vaccines and ICIs may offer a novel “death−clearance−activation” cooperative paradigm for next−generation cancer immunotherapy.

This review discusses the therapeutic applications of efferocytosis based on disease type (e.g., atherosclerosis, cancer, autoimmune diseases), analyzes various materials and intervention strategies for modulating efferocytosis, and reviews their mechanisms of action and preclinical outcomes. Through a structured framework, we aim to present the overall landscape and future potential of efferocytosis in disease treatment, and explore its cutting-edge applications in regenerative and precision medicine.

## 2 The fundamental mechanisms of efferocytosis and its role in tissue repair

To understand how efferocytosis contributes to tissue repair and disease treatment, it is essential to first elucidate its fundamental molecular mechanisms and physiological roles. This section provides an overview of the key steps involved in efferocytosis and discusses its dual functions in maintaining homeostasis and promoting repair under both normal and pathological conditions.

Efferocytosis is a vital biological process that removes apoptotic cells and maintains tissue homeostasis, primarily mediated by phagocytes such as macrophages and dendritic cells (10). During tissue repair, efferocytosis facilitates the clearance of necrotic cells and inflammatory mediators, promotes the release of anti-inflammatory signals, and regulates the immune microenvironment to accelerate healing (2).

**Figure 1:**
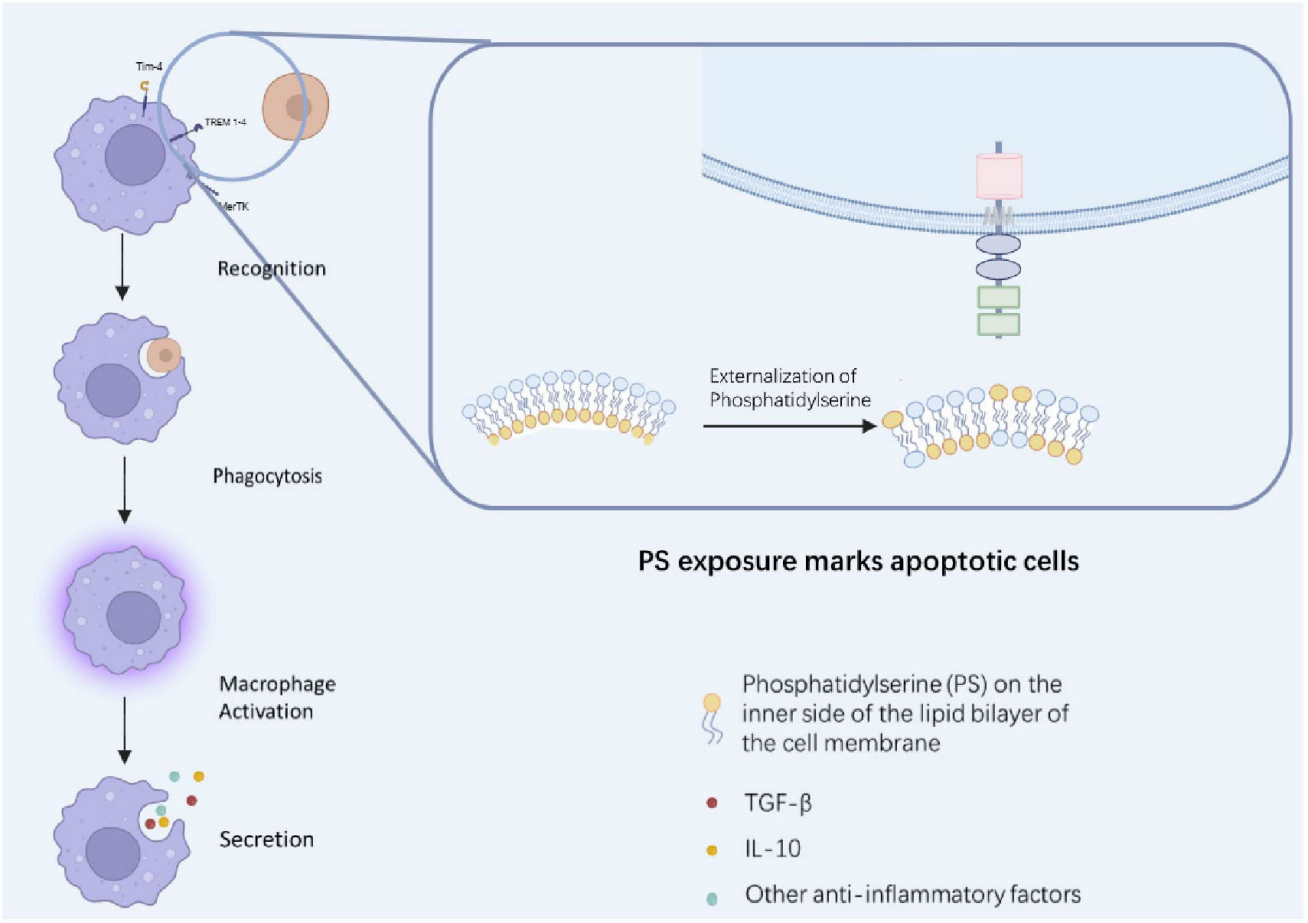
Mechanisms of efferocytosis and its immunomodulatory effects. Apoptotic cells externalize phosphatidylserine (PS) to the outer leaflet of their plasma membrane, serving as an “eat me” signal. This signal is recognized by specific receptors on macrophages, such as Tim-4, Axl, MerTK, and triggering receptors expressed on myeloid cells (TREM1-4). The recognition is often enhanced by bridging molecules like MFG-E8 and Gas6. This binding initiates the phagocytic engulfment of the apoptotic cell. Following engulfment, the macrophage undergoes a phenotypic switch, secreting anti-inflammatory cytokines (e.g., TGF-β, IL-10) and suppressing pro-inflammatory pathways (e.g., NF-κB, NLRP3 inflammasome). This cascade is crucial for resolving inflammation, promoting tissue repair, and maintaining immune homeostasis.

The basic process of efferocytosis involves three main stages: recognition, engulfment, and post-engulfment immune modulation (11). As shown in **Figure 1**, apoptotic cells expose “eat-me” signals, such as phosphatidylserine (PS), on their surface. Phagocytes recognize these signals through specific receptors (e.g., Mer tyrosine kinase (MerTK), Axl, T-cell immunoglobulin and mucin domain 4 (Tim-4)), often aided by bridging molecules like milk fat globule-epidermal growth factor 8 (MFG-E8) and growth arrest-specific 6 (Gas6), which then triggers the engulfment of the apoptotic cell (12, 13).

After engulfment, phagocytes enter an immunoregulatory phase, releasing anti-inflammatory cytokines such as TGF-β and IL-10, and suppressing pathways such as NF-κB and NLRP3 inflammasomes, thereby reducing inflammation and promoting tissue remodeling (14, 15). Specifically, the inhibition of NF-κB signaling diminishes the transcription of pro−inflammatory genes (e.g., TNF−α, IL−6), while NLRP3 inflammasome suppression limits caspase−1 activation and the subsequent maturation of IL−1β and IL−18. These coordinated changes shift the local milieu toward resolution and repair (16). Notably, apoptotic cells release extracellular vesicles, known as apoptotic vesicles (ApoEVs), which serve as key signal carriers during efferocytosis. These vesicles expose “eat-me” signals such as phosphatidylserine (PS) and calreticulin (CRT), enhancing their recognition and uptake by phagocytes. Once internalized, ApoEVs can deliver functional molecules (e.g., miRNAs, proteins) that further modulate macrophage polarization, suppress pro-inflammatory pathways, and activate regenerative programs, thereby deepening the mechanistic link between apoptotic clearance and tissue repair (17). Simultaneously, efferocytosis drives the metabolic and phenotypic transition of macrophages from a pro-inflammatory M1 state to a reparative M2 state (18, 19). However, this phenotypic switch is not absolute; some studies suggest that efferocytosis under chronic inflammatory conditions may sustain a hybrid or pro-inflammatory macrophage phenotype, complicating therapeutic strategies aimed at polarizing macrophages (20, 21). In addition to the direct immunomodulatory and reparative outcomes of efferocytosis, recent evidence highlights the convergence of efferocytosis with other cytoprotective signaling pathways, particularly those involving sirtuin (Sirt) family proteins. For example, melatonin, a pleiotropic hormone, has been shown to exert cardioprotective, anti-inflammatory, and antioxidative effects by activating Sirt1 and Sirt3 signaling pathways. Melatonin enhances Sirt1-mediated deacetylation of transcription factors like FOXO1 and NF-κB, thereby reducing oxidative stress, suppressing NLRP3 inflammasome activation, and promoting mitochondrial biogenesis and autophagy (22). These Sirt-driven mechanisms closely parallel the immunoresolutive and tissue-reparative functions of efferocytosis, suggesting potential cross-talk or synergistic interactions between efferocytic clearance and Sirt-regulated cellular homeostasis. Such integrative signaling networks may offer novel targets for combined therapeutic strategies aimed at amplifying tissue repair while modulating inflammatory responses across multiple organ systems (22).

Efferocytosis has demonstrated therapeutic effects in various tissues. For example, in skin injury, enhanced efferocytosis promotes necrotic cell clearance, shortens inflammation, and activates fibroblasts. In atherosclerosis, boosting macrophage clearance of foam cells helps inhibit plaque progression and repair the vascular endothelium (23, 24). In nervous system injuries, glial cell-mediated efferocytosis aids in clearing necrotic neurons, reducing neuroinflammation, and promoting axonal regeneration (25).

However, in pathological conditions such as chronic wounds, fibrosis, tumor microenvironments, and atherosclerosis, efferocytosis is often impaired, leading to apoptotic cell accumulation, chronic inflammation, and aggravated tissue damage (25, 26). In such contexts, exogenous or engineered apoptotic vesicles have emerged as promising therapeutic tools to restore efferocytosis and promote healing. For instance, MSC-derived ApoEVs have been shown to enhance osteogenic differentiation and bone regeneration by activating autophagy via Rab7 delivery (17). Similarly, apoptotic vesicles from young MSCs can rejuvenate aged stem cells and promote cartilage repair through modulation of macrophage polarization and inflammatory signaling (17). These examples illustrate how ApoEVs can be harnessed to amplify the natural regenerative outcomes of efferocytosis. Thus, restoring or enhancing efferocytosis has become a critical strategy for intervening in chronic diseases and promoting tissue regeneration. In recent years, regenerative medicine platforms based on nanomaterials, hydrogels, and 3D scaffolds designed to promote efferocytosis have become a research hotspot.

Given its core repair function, increasing efforts have focused on developing smart materials that mimic efferocytosis mechanisms. These systems not only efficiently clear necrotic cells but also precisely regulate local immune responses and activate endogenous repair programs. Therefore, efferocytosis-driven therapeutic platforms are emerging as promising strategies for tissue regeneration and immunotherapy.

## 3 Construction of efferocytosis-driven therapeutic platforms

Building on the foundational mechanisms of efferocytosis, researchers have developed various material-based platforms to harness its therapeutic potential. This section reviews recent advances in the design and application of nanomaterials, hydrogels, and biological scaffolds for modulating efferocytosis in different disease contexts.

With the rapid development of nanotechnology, biomaterials, and tissue engineering, the regulation of efferocytosis is gradually transitioning from basic research to precise therapeutic practice. To effectively activate or block the efferocytosis process, researchers have developed a series of targeted, biocompatible, and functionally responsive material platforms, aiming to simulate, activate, or reprogram the interaction between phagocytes and apoptotic cells. Based on the therapeutic needs in different disease models, these material strategies can be roughly categorized into three types: nanomaterial platforms, hydrogel systems, and biological scaffolds with hybrid materials. Each material intervenes in the efferocytosis process through different mechanisms, offering unique biological functions and application advantages.

### 3.1 Nanomaterial platforms: pioneers in precision delivery and immune regulation

Nanomaterials have emerged as versatile tools for modulating efferocytosis due to their tunable physicochemical properties and ability to interface with biological systems. The following subsections highlight their applications in cardiovascular diseases, cancer immunotherapy, and autoimmune disorders.

Nanomaterials are widely used to regulate efferocytosis because their small size and modifiable surfaces enable drug delivery, signal modulation, and immune remodeling. Researchers design biomimetic nanoparticles to either target macrophages, mimic “eat-me” signals, or reprogram immune cells, all of which can enhance efferocytosis. These strategies have made significant progress in the fields of atherosclerosis, cancer immunotherapy, autoimmune diseases, and other disease areas.

#### 3.1.1 Nanomaterials in cardiovascular diseases (such as atherosclerosis, myocardial infarction)

Cardiovascular diseases, particularly atherosclerosis, are characterized by impaired clearance of apoptotic cells and chronic inflammation. Nanomaterial-based strategies aimed at enhancing efferocytosis have shown promise in stabilizing plaques and promoting vascular repair.

Atherosclerosis is a chronic inflammatory disease characterized by foam cell accumulation and necrotic core formation. Its development is closely related to macrophage clearance of apoptotic cells (27, 28). Efferocytosis dysfunction is one of the core mechanisms of lesion progression, leading to apoptotic cell accumulation, inflammation, and plaque formation instability (29, 30). Therefore, restoring macrophage clearance function becomes a key therapeutic target.

Currently, major methods to enhance efferocytosis include the following three categories: first, by mimicking macrophage membrane surfaces, enhancing macrophage clearance by competing with non-specific targets; second, by mimicking apoptotic cell surface characteristics, increasing the recognition signal concentration for macrophages, thus promoting efferocytosis activation; and third, by loading bioactive molecules or drugs to target and activate macrophage efferocytosis pathways, further enhancing their immune clearance function. Researchers have developed various nanomaterials to enhance efferocytosis and inhibit the progression of atherosclerosis.

In 2022, Sha et al. developed macrophage membrane-camouflaged nanoparticles that enhanced efferocytosis and foam cell clearance, reducing plaque formation and inflammation (4). Chen et al. utilized platelet membrane-coated nanocarriers to deliver anti-CD47 antibodies, effectively blocking the “don’t eat me” signal axis and improving macrophage phagocytosis (26). Bao et al. designed engineered neutrophil apoptotic bodies that targeted infarcted myocardium, induced macrophage efferocytosis, and promoted myocardial tissue repair (31).

In 2023, He et al. reported a biomimetic nanozyme material with both anti-inflammatory and pro-efferocytic functions, which stabilized plaques and facilitated apoptotic cell clearance (32). In 2024, Cheng et al. developed a piezocatalytic “Trojan horse” nanoplatform that released drugs in response to local stimuli and activated macrophage efferocytosis to improve vascular permeability (33). Patel et al. proposed nanoparticles that induced M2 macrophage polarization and possessed dual functions of inflammation resolution and efferocytosis activation (34).

Han et al. constructed apoptotic cell-mimicking liposomes that activated macrophage efferocytosis, enabling targeted drug delivery and inflammation reduction (35). In 2025, Zhang et al. created a black phosphorus nanosheet-based therapeutic platform that scavenged reactive oxygen species (ROS), suppressed lipid peroxidation, and activated efferocytosis to reduce plaque area and inflammatory cytokine levels such as TNF-α and IL-6 (36). Tang et al. developed an HDL-modified nanodrug system that simulated natural HDL functions, promoted cholesterol efflux, and activated efferocytosis to remove necrotic cells and mitigate foam cell formation, effectively alleviating atherosclerotic lesions (37).

These findings indicate that nanomaterials for atherosclerosis therapy integrate anti-inflammatory, antioxidative, lipid-clearing, and vascular-protective functions. By remodeling the atherosclerotic microenvironment through efferocytosis, they achieve efficient clearance of apoptotic cells, suppression of inflammation, and plaque stabilization, offering innovative strategies for precision cardiovascular treatment.

A summary of recent studies employing nanomaterial-based strategies to modulate efferocytosis in cardiovascular and inflammatory disease models is presented in **Table 1**. These studies highlight how different nanomaterial platforms—ranging from biomimetic particles to bioactive nanosheets—can enhance phagocyte function, promote immune clearance, and support tissue regeneration through efferocytosis activation.

**Table 1:** Summary of nanomaterial applications in efferocytosis regulation.

Researchers	Nanomaterial type	Target disease	Key findings
Sha et al. (4)	Macrophage membrane biomimetic nanoparticles	Atherosclerosis	Enhanced efferocytosis, reduced plaque formation, and inflammation inhibition
Chen et al. (26)	Platelet membrane-coated nanocarriers	Atherosclerosis	Increased macrophage phagocytosis by blocking “don’t eat me” signals, enhanced plaque clearance
Bao et al. (31)	Engineered neutrophil apoptotic bodies	Myocardial infarction	Targeted infarct regions, induced macrophage efferocytosis, promoted tissue repair
He et al. (32)	Biomimetic nanozyme materials	Atherosclerosis	Anti-inflammatory and efferocytosis-promoting properties that stabilize plaques
Cheng et al. (33)	Piezocatalytic nanomaterial system	Atherosclerosis	Activated macrophage efferocytosis and improved vascular permeability under local stimulation
Patel et al. (34)	Nanoparticles inducing M2 macrophage polarization	Atherosclerosis	Dual pro-inflammatory resolution and efferocytosis activation properties for effective plaque treatment
Han et al. (35)	Biomimetic liposomes	Inflammation	Activated efferocytosis pathways, improved drug delivery efficiency, and reduced local inflammation
Zhang et al. (36)	Black phosphorus nanosheets	Atherosclerosis	Targeted macrophage efferocytosis, reduced plaque size, and lowered inflammatory cytokines
Tang et al. (37)	HDL-modified nanomedicines	Atherosclerosis	Enhanced cholesterol clearance and efferocytosis activation to slow foam cell formation

This table summarizes recent advances in the use of nanomaterials for enhancing or reprogramming efferocytosis, particularly in models of atherosclerosis, myocardial infarction, and inflammation. Various strategies include mimicking apoptotic cell surfaces, blocking “don’t eat me” signals, loading efferocytic modulators, or inducing macrophage polarization. These approaches demonstrate promising outcomes in reducing plaque burden, promoting tissue repair, and regulating immune microenvironments.

#### 3.1.2 Applications in cancer immunotherapy

In the context of cancer, efferocytosis plays a dual role: while it can help clear tumor cells, it may also contribute to immunosuppression. Nanomaterial-based approaches that modulate efferocytosis offer new avenues for enhancing antitumor immunity.

In the tumor microenvironment, macrophages play a vital role in efferocytosis, influencing immune responses and tumor progression (38, 39). Notably, the therapeutic goal of modulating efferocytosis in cancer often contrasts with that in cardiovascular diseases such as atherosclerosis. In atherosclerosis, strategies primarily aim to enhance efferocytosis to clear apoptotic cells (e.g., foam cells), thereby reducing inflammation and stabilizing plaques. In contrast, within established tumors, efferocytosis by tumor-associated macrophages (TAMs) frequently supports an immunosuppressive microenvironment by releasing anti-inflammatory signals (e.g., TGF-β, IL-10) and promoting regulatory T cell recruitment. Therefore, in cancer immunotherapy, a key strategy is to block or reprogram this pathological efferocytosis to reverse immunosuppression, enhance antigen exposure, and reinstate antitumor immunity.

Recent cutting−edge studies indicate that modulating efferocytosis alone may be insufficient to achieve durable antitumor immunity. Instead, triple−combination strategies that integrate immune checkpoint blockade (e.g., anti−PD−1, anti−CTLA−4 antibodies) with therapeutic cancer vaccines can produce synergistic effects: vaccines provide tumor−specific antigens, efferocytosis regulation clears apoptotic cells and alleviates local immunosuppression, and checkpoint inhibitors unleash pre−existing T−cell functions (9). Such combinatorial approaches have demonstrated superior tumor control and survival benefits over monotherapies in preclinical models. For instance, GM−CSF−secreting tumor cell vaccine (GVAX) combined with anti−PD−1 antibody significantly prolonged survival in murine colon cancer models; the personalized neoantigen vaccine NEO−PV−01, when administered with pembrolizumab (anti−PD−1), induced durable neoantigen−specific CD4^+^ and CD8^+^ T−cell responses in patients with non−small cell lung cancer (9). These findings suggest mechanistic crosstalk and complementarity among efferocytosis, vaccination, and checkpoint blockade, and their integrated application may help overcome current limitations in cancer immunotherapy. Dysfunctional efferocytosis facilitates tumor immune evasion and promotes an immunosuppressive state. Nevertheless, the role of efferocytosis in cancer is paradoxical: while it can clear apoptotic cells and reduce inflammation, excessive efferocytosis in established tumors may enhance immune suppression and promote tumor progression by releasing anti-inflammatory signals and growth factors (40). This duality necessitates careful modulation—either enhancing or suppressing efferocytosis—to reprogram the tumor microenvironment, restore immune activation, and improve immunotherapeutic outcomes (41). Modulating efferocytosis—either enhancing or suppressing it—can reprogram the tumor microenvironment, restore immune activation, and improve immunotherapeutic outcomes (8, 42).

In 2023, Zhuang et al. constructed a multifunctional nanosystem based on bacterial outer membrane vesicles that blocked efferocytosis and activated tumor-specific immunity, significantly inhibiting tumor growth (5). Chen et al. developed membrane-coated nanocomposites that mimicked tumor membranes, enhanced immune recognition, and blocked efferocytosis while activating the cGAS/STING pathway, thereby extending survival in melanoma models and enhancing PD-1 inhibitor efficacy (43). Zhang et al. co-delivered doxorubicin and BMS777607 via tailored nanoparticles to simultaneously block efferocytosis and boost immune responses, enhancing chemotherapy outcomes (44).

Shang et al. designed engineered nanospores that selectively blocked LC3-associated phagocytosis in tumor-associated macrophages, thus potentiating antitumor immunity (45). Wu et al. proposed “efferocytosis nanoinhibitors” that promoted secondary necrosis to enhance antigen release and improve the immunogenicity of radiotherapy and chemotherapy (46).

In 2024, Cheng et al. revealed a CD276-mediated efferocytosis mechanism enabling immune evasion in bladder cancer, suggesting a potential immunotherapy target (47). Zhou et al. used RNA-based strategies to modulate macrophage efferocytosis, improving antigen presentation and T-cell activation in colorectal cancer (48). Li et al. developed erythrocyte-mimicking nanocarriers that delivered photoactivated STING agonists to reprogram tumor-associated macrophages, stimulate antitumor immunity, and reduce recurrence (49).

These studies demonstrate that modulating efferocytosis is key to reversing immunosuppression in tumors. By blocking aberrant efferocytosis, it is possible to activate effective antitumor immune responses and improve cancer immunotherapy.

#### 3.1.3 Applications in autoimmune disease therapy

Autoimmune diseases often involve defective clearance of apoptotic cells, leading to exposure of self-antigens and chronic inflammation. Nanomaterials that enhance efferocytosis can help restore immune tolerance and mitigate autoimmune pathology.

In autoimmune diseases, delayed clearance of apoptotic cells can expose intracellular antigens, leading to autoimmune responses and sustained inflammation (50, 51). Efferocytosis dysfunction results in apoptotic cell accumulation and immune dysregulation, making its restoration essential for maintaining immune tolerance (2).

Recent studies have employed biomimetic nanomaterials to reshape inflammatory microenvironments, induce immune tolerance, and reconstruct efferocytic pathways. In 2022, Yang et al. developed efferocytosis-inspired nanomaterials that reprogrammed synovial macrophages, alleviating cartilage degradation and reducing inflammatory cytokines in osteoarthritis (52). In 2024, Zhang et al. created biomimetic nanoparticles mimicking apoptotic cell membranes, which reprogrammed macrophage phenotypes in a rheumatoid arthritis mouse model, enhanced immune tolerance, and reduced IL-1β and IL-17 expression, thus relieving joint inflammation and preventing tissue damage (6).

These findings highlight the crucial role of efferocytosis in autoimmune regulation. From rheumatoid arthritis to osteoarthritis, enhancing or restoring efferocytic function can alleviate inflammation and restore immune homeostasis. Remodeling the efferocytosis pathway offers a dual therapeutic strategy for apoptotic cell clearance and immune modulation. In this regard, apoptotic vesicles have demonstrated direct immunomodulatory capacity. For example, ApoEVs derived from T cells or MSCs can suppress Th17 differentiation and promote regulatory T cell (Treg) expansion via TGF-β signaling, thereby ameliorating experimental colitis and rheumatoid arthritis (17). Moreover, ApoEVs carrying miR-21-5p can reprogram M1 macrophages toward an M2 phenotype, reducing synovial inflammation and joint damage in osteoarthritis (17). These mechanisms underscore how ApoEVs can be designed to precisely modulate immune responses within the efferocytosis framework.

### 3.2 Hydrogel systems: scaffolds for modulating the efferocytic microenvironment and tissue regeneration

Hydrogels provide a three-dimensional and hydrated environment that can be tailored to mimic native tissue conditions. Their versatility makes them ideal platforms for delivering efferocytosis-modulating signals and cells in a spatially and temporally controlled manner.

As research on efferocytosis continues to deepen, hydrogel systems have emerged as both “smart delivery platforms” and “immune microenvironment modulators” in regenerative medicine, chronic wound repair, and immune-related disease therapy. Recent studies suggest that hydrogels, by mimicking the dynamic processes of apoptosis, signal release, and phagocytic clearance, can effectively regulate inflammatory responses, activate repair-related cells, and even stimulate antitumor immunity.

In chronic inflammatory conditions such as degenerative diseases, non-healing wounds, and periodontitis, hydrogels allow for the precise delivery of immunomodulatory drugs or signaling molecules that reprogram local immune cell behavior. In cancer therapy, hydrogels designed to mimic efferocytic mechanisms have enhanced the immune system’s ability to recognize and eliminate tumor antigens, opening new pathways for immunotherapeutic strategies.

**Figure 2:**
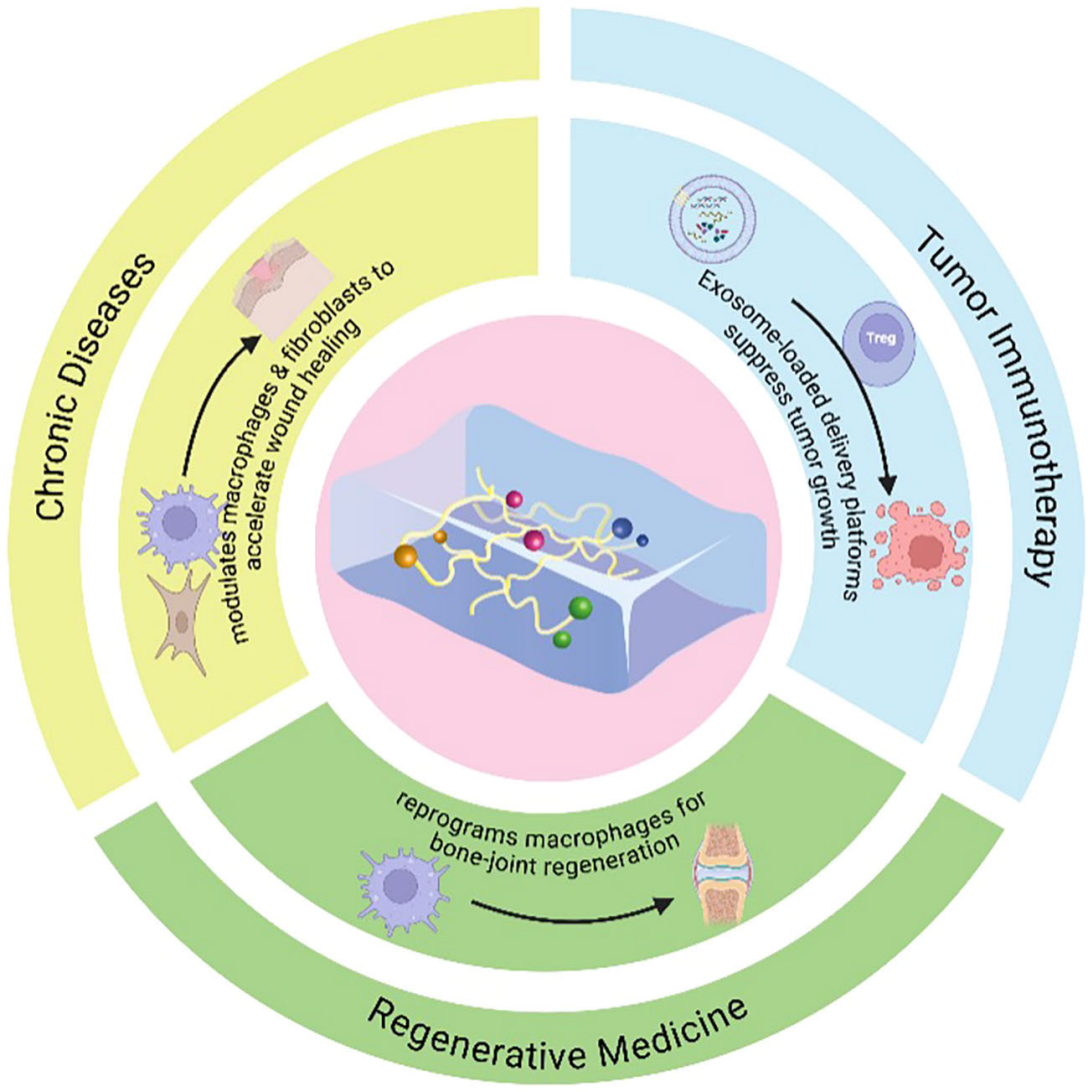
Schematic diagram of the application of hydrogel-regulated efferocytosis in regenerative medicine, tumor immunology and chronic disease treatment. Hydrogel systems are designed to regulate macrophage polarization, promote the clearance of apoptotic or senescent cells, and modulate the local immune environment. In chronic wounds, hydrogels accelerate healing by regulating efferocytosis and fibroblast activity. In tumor immunotherapy, hydrogels serve as delivery platforms to enhance immune cell activation and antigen presentation. In regenerative medicine, they support joint and cartilage repair by inducing macrophage-mediated efferocytosis and anti-inflammatory signaling. This multifunctional strategy positions hydrogels as key tools for coordinating immune clearance and tissue regeneration.

As illustrated in **Figure 2**, hydrogel systems influence multiple aspects of efferocytosis, including macrophage polarization, apoptotic cell clearance, and immune signal transmission. In models of chronic wounds, bone and joint degeneration, and cancer immunotherapy, these hydrogels have successfully constructed locally controllable efferocytic microenvironments, thereby enhancing both tissue repair and immune response. This dual functionality—structural support and immune signal modulation—positions hydrogels as highly versatile therapeutic platforms.

For example, Lee et al. (53) developed a thermosensitive hydrogel loaded with CD146/IGF-1 nanoparticles, which promoted skeletal muscle regeneration by activating efferocytosis and shifting immune responses toward an M2-like repair phenotype. Liu et al. (54)constructed a hybrid biomimetic hydrogel that synchronously activated early inflammatory responses and later anti-inflammatory regulation, thereby promoting effective healing of refractory wounds.

Yang et al. (55) proposed an inflammation-responsive sprayable hydrogel designed to prevent trauma-induced heterotopic ossification by locally modulating efferocytic activity and maintaining immune homeostasis. Li et al. (56) encapsulated engineered exosomes in an injectable hydrogel to simulate “injured cell–phagocyte” communication, enhancing antigen presentation and T cell activation while significantly inhibiting tumor growth.

In joint degeneration models, Xiong et al. (57) designed hydrogel microspheres capable of site-specific signal release to reverse chondrocyte senescence and polarize macrophages toward an M2 phenotype, thereby delaying cartilage degeneration. Jin et al. (58) developed a hydrogel platform with stepwise regulatory capabilities that modulated macrophage–fibroblast interactions to accelerate diabetic wound healing.

In osteoarthritis therapy, Wang et al. (59) developed pro-efferocytic microspheres within a hydrogel matrix to promote M2 polarization of macrophages, thus facilitating synovial remodeling and cartilage repair. Zhuo et al. (60) used a GelMA hydrogel to deliver a neutrophil apoptosis inducer, effectively reducing periodontitis-related inflammation and promoting alveolar bone regeneration. He et al. (61) constructed a dual-delivery hydrogel system incorporating exosomes and AAV vectors to simulate efferocytic signaling cascades, promoting vascularization and collagen deposition in diabetic wounds. Notably, the immunomodulatory and pro-regenerative effects of hydrogels can be synergistically enhanced by integrating components derived from perinatal tissues, which are rich sources of bioactive factors and stem cells. For instance, human amniotic membrane (AM) and its derivatives have been extensively used in dermatology for wound healing, scar reduction, and treatment of inflammatory skin conditions like psoriasis and atopic dermatitis (62, 63). The AM and its conditioned medium (CM) contain a multitude of anti-inflammatory cytokines (e.g., IL-10, IL-1RA), growth factors (e.g., EGF, FGF, TGF-β3), and extracellular matrix components that collectively promote macrophage polarization toward an M2 phenotype, suppress neutrophil infiltration, and enhance keratinocyte/fibroblast proliferation—processes that are intrinsically linked to efficient efferocytosis and inflammation resolution (62). Similarly, hydrogels loaded with umbilical cord mesenchymal stem cell (UC-MSC) derived exosomes or conditioned media have demonstrated accelerated diabetic wound closure and reduced scarring by modulating the PI3K/AKT/mTOR pathway and downregulating pro-fibrotic TGF-β1 (63). These examples underscore how the principles of efferocytosis—clearing apoptotic debris and switching the immune milieu from pro-inflammatory to pro-repair—are actively harnessed by perinatal tissue derivatives. Incorporating such bioactive elements into hydrogel designs offers a promising strategy to create “efferocytosis-mimetic” microenvironments that can robustly guide tissue regeneration.

**Figure 2** and **Table 2** summarize these applications, highlighting the diversity and therapeutic potential of hydrogel-based systems integrated with efferocytosis mechanisms. These systems combine precise cell clearance with local immune regulation, offering new, more efficient treatment options across a range of disease models.

**Table 2:** Summary of research on hydrogel materials in efferocytosis mechanisms.

Researchers	Research focus	Therapeutic strategy	Key intervention	Results
Lee et al. (53)	Muscle regeneration repair	Thermoresponsive hydrogel loaded with CD146/IGF-1 nanoparticles to promote muscle regeneration and immune regulation	Thermoresponsive hydrogels modulate efferocytosis and immune regulation to promote muscle regeneration	Significantly repaired skeletal muscle defects and restored muscle function
Liu et al. (54)	Non-healing wound repair	Hybrid biomimetic materials that activate local inflammation and efferocytosis to initiate wound repair	Hybrid biomimetic hydrogels induce local inflammation and efferocytosis, triggering wound repair	Effectively initiated the repair of non-healing wounds, significantly reducing inflammation and promoting tissue healing
Yang et al. (55)	Suppression of heterotopic ossification	Inflammation-responsive spray hydrogel system to suppress trauma-induced heterotopic ossification via efferocytosis regulation	Spray hydrogel system modulates immune cells to prevent trauma-induced heterotopic ossification	Effectively prevented trauma-induced heterotopic ossification and suppressed abnormal calcification processes
Li et al. (56)	Cancer immunotherapy	Engineered exosome-loaded injectable hydrogels to simulate “damaged cell-phagocyte” signaling, enhancing antigen presentation and T-cell activation	Exosome-loaded hydrogels enhance antigen presentation and T-cell activation in cancer models	Significantly inhibited tumor growth and enhanced immune response
Xiong et al. (57)	Degenerative joint disease	Microsphere hydrogels for lesion-specific drug delivery to induce macrophage polarization and cartilage regeneration	Microsphere hydrogels release apoptotic cell and macrophage polarization factors at the lesion site	Improved cartilage degeneration, slowed osteoarthritis progression, and restored joint function
Jin et al. (58)	Diabetic wound healing	Multi-level efferocytosis-regulated hydrogel platform to promote wound healing through macrophage-fibroblast interactions	Hydrogel regulates macrophage-fibroblast interactions to accelerate diabetic wound healing	Accelerated wound closure and improved tissue remodeling in diabetic mice
Wang et al. (59)	Joint repair in osteoarthritis	*In situ* pro-efferocytosis microsphere hydrogels to promote macrophage M2 polarization for joint repair	Microsphere hydrogels modulate macrophage polarization and improve joint inflammation	Significantly improved cartilage repair, reduced joint inflammation, and restored function in osteoarthritis models
Zhuo et al. (60)	Periodontal bone loss	Gelatin methacryloyl (GelMA)-based hydrogels delivering neutrophil apoptosis inducers to modulate immune responses	GelMA hydrogels modulate neutrophil apoptosis and macrophage M2 polarization for periodontal bone regeneration	Promoted alveolar bone regeneration and reduced periodontal inflammation in animal models
He et al. (61)	Diabetic wound healing	Dual-carrier hydrogel system (exosomes + adeno-associated virus) to promote vascularization and collagen deposition in diabetic wounds	Dual-carrier hydrogels regulate immune microenvironment, promote vascularization, and enhance wound healing	Improved blood vessel formation, collagen deposition, and accelerated diabetic wound healing

This table summarizes current studies on hydrogel systems designed to regulate efferocytosis and modulate local immune responses across various disease models, including diabetic wounds, joint degeneration, cancer, and periodontitis. By delivering immunoregulatory cues, promoting macrophage polarization, or simulating apoptotic signaling, these hydrogels demonstrate potent regenerative and anti-inflammatory effects, highlighting their translational potential in precision medicine.

From the studies above, it is evident that hydrogel systems possess three key functions—biomechanical support, biological signal delivery, and local immune modulation—which make them ideal platforms for efferocytosis-based therapies. Whether for diabetic wound healing, osteoarthritic degeneration, or cancer immunotherapy, hydrogels can modulate the entire loop of “cell recognition–phagocytosis–inflammation resolution–tissue regeneration.” Looking forward, combining hydrogels with gene editing, engineered exosomes, or intelligent nanomedicines may further propel efferocytosis-based treatments toward personalized and precision medicine.

### 3.3 Biological scaffolds and hybrid systems: multi-modal efferocytosis activation in tissue engineering

Biological scaffolds and hybrid systems represent another frontier in efferocytosis modulation, offering structural support and bioactivity to guide tissue regeneration. This subsection discusses how such platforms integrate efferocytic signals to promote repair in complex tissue environments.

Biological scaffolds and hybrid materials offer dual functions: they provide structural support for tissue engineering and act as carriers for efferocytosis-modulating components, such as signaling molecules, engineered exosomes, or biomimetic interfaces. By integrating these components, scaffolds can achieve multi-modal regulation of immune clearance and regenerative processes. This strategy offers new solutions for long-term implantation and complex tissue defect repair, promoting the deep integration of regenerative medicine and immune modulation.

In 2019, Lee et al. (64) first proposed that fibronectin could serve as a signaling scaffold for Tim-4-mediated efferocytosis. They demonstrated that incorporating efferocytic ligands into scaffold materials enhanced macrophage phagocytic function and promoted tissue regeneration. This finding revealed the intrinsic link between efferocytic signaling and scaffold-mediated tissue repair, providing a novel direction for functional scaffold design.

In 2021, Gerlach et al. (65) further showed that efferocytosis not only eliminates apoptotic cells but also induces macrophage proliferation, facilitating reparative immune activation and accelerating tissue remodeling. This expanded the conceptual role of efferocytosis in regeneration by highlighting its impact on immune cell fate decisions.

Building on this concept, Zou et al. (66) in 2022 developed a pro-efferocytic exosome-loaded vascular stent that responds to Lp-PLA_2_ enzymatic activity. This system enables targeted exosome release, enhances local macrophage clearance of necrotic cells, reduces inflammation, and significantly lowers restenosis rates while improving stent stability. The study demonstrated the feasibility and translational value of integrating efferocytosis into vascular biomaterial engineering.

These studies underscore that biological scaffolds incorporating efferocytic functionality can achieve simultaneous optimization of structural reconstruction and immune regulation. By precisely designing scaffold surface properties or incorporating functional components, such materials not only enhance macrophage clearance efficiency but also shape a microenvironment conducive to tissue repair. This approach provides both theoretical and practical support for developing the next generation of tissue engineering scaffolds with dual “immune activation–tissue guidance” capabilities. The concept of designing scaffolds to actively promote efferocytosis is further exemplified by the clinical success of decellularized perinatal tissues. Decellularized human amniotic membrane (dHAM) and Wharton’s jelly extracellular matrix (dWJ-ECM) have been used as wound dressings and scaffolds in skin regeneration, showing remarkable abilities to reduce inflammation, prevent fibrosis, and promote vascularization (62, 63). Their efficacy is attributed not only to their structural support but also to their reservoir of retained immunomodulatory molecules (e.g., IL-10, TIMPs, HA) that facilitate apoptotic cell clearance and macrophage reprogramming. For instance, dHAM has been shown to downregulate IL-1β and TGF-β pathways while upregulating anti-inflammatory IL-10, thereby creating a local environment conducive to efferocytosis and scarless healing (62). Moreover, placental disc-derived ECM scaffolds rich in fibronectin and growth factors have been shown to enhance melanocyte migration and attachment in vitiligo treatment, a process that involves the clearance of stressed melanocytes and regeneration of pigmentation—a form of tissue repair linked to efferocytic mechanisms (63). These clinical and pre-clinical applications demonstrate that biological scaffolds naturally rich in efferocytosis-promoting signals can be powerful platforms for tissue engineering, providing a blueprint for the design of synthetic or hybrid scaffolds with similar immunomodulatory capabilities.

Although research on efferocytosis-modulating biological scaffolds is still in its early stages compared to nanomaterials and hydrogels, their unique ability to provide long-term structural support while enabling localized immune regulation presents unparalleled potential. This makes them particularly promising for addressing complex regenerative challenges, especially in the repair of bone, cartilage, and other structured tissues where mechanical integrity and biological signaling must be seamlessly integrated.

## 4 Prospects and challenges

While significant progress has been made in understanding and harnessing efferocytosis for therapeutic purposes, several challenges remain. This section outlines the future directions and potential obstacles in translating efferocytosis-based strategies into clinical practice.

### 4.1 Application prospects

As a core mechanism linking cell death to tissue regeneration, efferocytosis is now transitioning from fundamental research to clinical translation. Especially in fields such as tissue regeneration, immune modulation, and cancer therapy, efferocytosis-based strategies—integrated with advanced material technologies such as hydrogels, nanomaterials, and biological scaffolds—are showing multidimensional therapeutic potential. At present, hydrogels and similar materials, due to their excellent tunability, biocompatibility, and carrier properties, have been widely applied in chronic disease models including diabetic wound healing, degenerative joint diseases, and cancer immunotherapy, with encouraging outcomes.

In the future, with continued advancements in materials science and bioengineering, stimuli-responsive smart materials (e.g., photosensitive, thermosensitive, or enzyme-responsive hydrogels) are expected to enable more precise and personalized regulation of efferocytosis. Encouragingly, several pharmacological strategies targeting efferocytosis pathways have entered early-phase clinical trials, primarily in oncology. For instance, inhibitors of the “don’t eat me” signal CD47 (e.g., magrolimab) and agonists of the pro-efferocytic receptor MerTK are under evaluation for solid tumors and hematologic malignancies (67–69). Early trial data suggest potential for reshaping the tumor immune microenvironment, although efficacy varies and immune-related adverse events remain a concern (70). These clinical efforts validate the therapeutic relevance of modulating efferocytosis and pave the way for future translation of more sophisticated material-based platforms. Embedding bioactive components such as cells or extracellular vesicles (e.g., exosomes) into material systems may allow for multi-pathway synergistic modulation of efferocytic processes, thereby improving therapeutic efficacy and safety in complex disease contexts. These innovations are likely to accelerate the shift of efferocytosis from an “auxiliary mechanism” to a “therapeutic axis” in clinical medicine.

Moreover, the integration of efferocytosis−modulating platforms with advanced gene−delivery technologies offers a promising synergistic strategy for immune reprogramming and tissue regeneration. Chemical transfection/transduction enhancers—such as polycations, histone deacetylase inhibitors and small molecules—can significantly improve the efficiency of viral vector−mediated gene delivery, a key approach in the treatment of inherited retinal diseases and other genetic disorders (71). When combined with efferocytosis−focused biomaterials (e.g., hydrogels presenting “eat−me” signals or delivering apoptotic vesicles), these enhancers may potentiate the localized expression of immunomodulatory or pro−reparative genes, thereby enabling more precise and sustained remodeling of inflammatory and degenerative microenvironments. Such combinatorial “gene−delivery + efferocytosis−activation” approaches could enhance therapeutic outcomes in chronic inflammatory diseases, degenerative conditions and cancer immunotherapy (71).

Furthermore, the field can draw inspiration from the rapid translation of perinatal tissue-derived products (e.g., dehydrated amniotic/chorionic membranes, umbilical cord biologics) for dermatological and inflammatory conditions (62, 63). These products have already demonstrated safety and efficacy in modulating local immune responses and promoting regeneration in humans. By dissecting the specific efferocytosis-related pathways activated by these biologics (e.g., MerTK/Axl signaling, anti-inflammatory cytokine release, macrophage polarization), researchers can identify key molecular targets and bioactive factors to incorporate into synthetic material systems. This convergence of insights from clinical regenerative medicine and fundamental efferocytosis biology will accelerate the development of precisely controlled, next-generation therapeutic platforms with enhanced regenerative potential.

### 4.2 Key challenges

Despite promising achievements in preclinical models, the broad clinical application of efferocytosis-regulating strategies still faces several critical challenges. First, the transition from promising preclinical data to human therapies is hindered by a lack of clinical trial evidence for advanced biomaterial systems. While small molecule and antibody approaches are in early trials, nanomaterials, hydrogels, and scaffolds—despite their sophisticated design—have not yet entered clinical testing, highlighting a significant translational gap.

Second, the precise coupling between materials and efferocytic mechanisms remains underdeveloped. Key bottlenecks include controlling *in vivo* degradation rates, drug release kinetics, spatial localization, and achieving specific interactions with immune cells. Specifically, the degradation profiles of nanomaterials and hydrogels—whether through hydrolysis, enzymatic action, or other mechanisms—must be finely tuned to match the therapeutic timeline. Too rapid degradation may curtail the sustained release of efferocytosis-modulating signals, while overly slow degradation or non-degradable components could lead to material accumulation, chronic inflammation, or fibrosis (72, 73). Although many systems rely on external stimuli or endogenous signals to activate efferocytosis, the controllability and stability of these responses within physiological environments remain to be optimized. Furthermore, the complexity of biomaterials raises unique safety concerns for clinical translation, such as long-term biocompatibility, unintended immune modulation, and potential off-target effects, which require rigorous evaluation under Good Manufacturing Practice (GMP) standards (74, 75). Of particular relevance is the inherent immunogenicity of some material components (e.g., certain synthetic polymers, residual endotoxins, or animal-derived proteins), which might provoke unintended innate or adaptive immune responses. Such responses could not only compromise material safety but also directly counteract the desired anti−inflammatory and pro−resolutive effects central to efferocytosis−based therapies (76).

Third, efferocytosis is an inherently balanced immune process. Excessive activation may lead to immune escape or tissue damage, whereas insufficient activation may fail to support clearance and repair. Therefore, material design must consider the temporal–spatial–quantitative dimensions of efferocytosis to ensure dynamic coordination between immune activation and tissue regeneration.

Fourth, the significant heterogeneity of the immune microenvironment across different patients and disease stages presents a major challenge for developing universal efferocytosis-modulating therapies. A material strategy that is effective in one context might be ineffective or even detrimental in another. This variability demands a shift towards personalized medicine approaches. Future research must focus on identifying predictive biomarkers (e.g., soluble MerTK, phosphatidylserine exposure levels) for patient stratification (35, 77) and developing “smart” platforms that can sense and adapt to the local pathological state, moving toward personalized efferocytosis modulation.

Lastly, there are significant barriers to clinical translation, including differences between animal models and human pathophysiology, limited long-term efficacy and biosafety data, and challenges in clinical trial design and ethical approval. Advancing efferocytic material systems toward clinical use will require interdisciplinary collaboration, standardized evaluation frameworks, and well-defined translational pathways.

## 5 Conclusion

In summary, efferocytosis represents a critical biological process that bridges cell death and tissue regeneration. In particular, within the field of cancer immunotherapy, combining efferocytosis modulation with cancer vaccines and ICIs represents a promising multi−level, multi−target therapeutic framework for the next generation of immunotherapies (9). By employing smart biomaterials to precisely deliver vaccine antigens, locally tune efferocytic activity, and synergize with systemic checkpoint blockade, it may be possible to achieve comprehensive immune activation—from local microenvironment reprogramming to systemic antitumor immunity—ultimately advancing oncology toward more precise, personalized, and effective therapeutic paradigms. This review has outlined its mechanisms, highlighted its roles in various diseases, and discussed the latest material-based strategies for its therapeutic modulation. The integration of efferocytosis with advanced biomaterials holds great promise for next-generation therapies in regenerative and precision medicine.

By deeply integrating immunology, materials science, and clinical medicine, the “death-to-rebirth” strategy targeting efferocytosis is poised to revolutionize the treatment paradigm for a range of intractable diseases, moving beyond symptomatic relief towards true tissue restoration and immune reprogramming.

The application of efferocytosis in disease treatment is entering a critical phase, demonstrating powerful potential particularly in areas such as tissue repair, immune regulation, and cancer therapy. With the convergence of materials science, bioengineering, and immunology, the development of smart materials capable of modulating efferocytosis offers new therapeutic solutions for chronic diseases, degenerative conditions, and immune-related disorders.

However, to fully bridge the gap between basic research and clinical practice, several core challenges must be addressed, including the precision of material design, the controllability of immune responses, and the systematic planning of translational pathways. Future research should focus on the detailed dissection of efferocytosis regulatory mechanisms and the integrated optimization of material functionality, aiming to establish safer, more efficient, and personalized therapeutic systems. In doing so, efferocytosis-driven therapies will provide a broader clinical future for the concept of “life from death” in regenerative medicine.
